# An Investigation into the Effects of Cold upon the Body

**Published:** 1918-02

**Authors:** 


### *An Investigation into the Effects of Cold upon the Body.*

By Norman C. Lake, Temporary Captain, R. A. M. C. Abs. from the *Lancet,* Oct. 13, 1917.

The author reports upon a large number of experiments performed on isolated individual tissues, both animal and human, to determine the exact nature of the influence of cold in producing frost-bite. The method of growing tissues *in vitro* devised by Burrows and improved by Carrel was used in most of the experiments. Both tissues and blood were tested in this manner, and the author concludes that a temperature of $-6^{\circ}$ C. is necessary to produce absolute destruction of cells and freezing of blood. He therefore regards $-6^{\circ}$ C. as a temperature " critical in nature as far as the function and activity of both tissues and blood cells go ".

M. also carried out experiments to investigate the effects of cold upon the tissues in normal relationship to the circulatory and nervous systems. They were performed not only with animal but also with volunteer human subjects. From these experiments he concludes that $-6^{\circ}$ C. is also the critical temperature for tissues in normal relationship to the body.

Further tests were made upon animals and men to determine the effect of various abnormalities of vessels and nerves, such as nerve " blocking " and vaso-constriction. He concludes that true frostbite produced by a temperature lower than $-6^{\circ}$, since it consists in the destruction of tissues, cannot be entirely prevented by modifying either the blood or nerve supply to the part. In the case of " chilling " at temperatures above $-6^{\circ}$ C. blocking of the nerves does not prevent congestion although it makes it less severe. Congestion, however, may be prevented in such cases by the local injection of adrenalin, and to some extent by injections of cocaine.

The author discusses the results of his experiments from a theoretical point of view. He remarks that the temperature of $-6^{\circ}$ C. was in all his experiments the degree of cold required for tissue destruction. The change brought about was by no means gradual, as others have contended. The curves on all of the charts recordding the experiments, drop abruptly at this temperature, indicating almost immediate tissue change of a gross physical character. At a higher temperature, however, the author believes there may be chemical changes. He notes that upon his charts there is also a depression indicating loss of vitality at about the temperature of $+15^{\circ}$. This persistent phenomenon he explains by the assumption that the vital process of the cells, which may be summed up by the word metabolism, is first seriously interfered with at a temperature of about $15^{\circ}$ C. Metabolism, he contends, consists in a nice balance between anabolism and catabolism. At about $+15^{\circ}$ C., anabolism may be presumed to have stopped entirely, while catabolism continues slowly, until the temperature falls to 0. Not, however, until $-6^{\circ}$ C. is reached, do any physical changes take place.

In the case of actual frost-bite resulting from temperatures lower than $-6^{\circ}$ C., he considers the rapid exudation of fluids, of blood, and even of cells, one of the most important phenomena. It results from the destruction of the endothelial walls of capillaries, and invades the surrounding tissues. Thus pressure is created which may shut off the blood supply. This effect may also be produced by clotting due to the escape of tissue extracts. The author believes that the only means of treating the condition of actual frost-bite is to reduce this exudation. Shutting off the blood supply and blocking the nerves with cocaine appears to do no good. The local injection of cocaine, or better, of adrenalin, however, completely prevented the exudation in one or two cases, and in all cases it caused considerable delay and probably lessened the amount.

He explains this action of the adrenalin or cocaine injected locally on the assumption that the veins and capillaries, which have been interrupted, if not destroyed, by the action of the cold, are subjected suddenly upon thawing to high pressure from the larger arteries that have not been obstructed. The injection of the adrenalin or cocaine tends to constrict these vessels, thus lessening the pressure upon the damaged veins and capillaries during the readjusting period. If the damage to the capillary walls has not been extensive, all exudation may thus be prevented. As a further means of damming back the blood supply he thinks intravenous injections of vaso-constrictors would be justifiable during the period of the thaw. He has not, however, been able to employ this method, but thinks it worthy of trial. He admits that in really serious cases of actual frost-bite, nothing can be done.

The condition of chilling or "frigorism" as Delépine has called it, produced by temperatures above $-6^{\circ}$ C., cannot be explained, the author believes, on the ground of cold alone, and he again attributes the seriousness of the malady to exudation. Even the nervous disturbances noted in trench feet he attributes to interneural or intraneural exudation into the larger nerves. The exudation in this case is not brought about so much by damage to the tissues from cold directly as by very high blood pressure suddenly produced upon the capillaries by the release of the constriction caused by cold. The swelling thus produced tends further to constrict the veins, and so a vicious circle begins.

Any artificial compression due to puttees or shoes may add seriously to the evil effects of this process. The vaso-dilation appears to the author to be entirely local and not reflex in its nature, because the blocking of the nerves with cocaine does not prevent its occurrence.

By way of treatment, he again recommends that every means be employed to keep the capillary pressure low while the arterioles regain their tone. Prophylaxis, he believes, should aim to prevent all artificial constriction. The maintenance of a thin layer of dry air by rubber boots or Delépine's waterproof stockings, he thinks, may help to avoid a rapid fall in temperature, which is likely to be more paralysing to the arterioles than constant cold. He does not believe, however, that water is ever an important cause of the condition of trench feet, because osmotic action could hardly occur through the thick protective skin of the feet.

*Treatment.* — " Once the limb has become definitely " chilled " treatment should be immediately started. It would appear better to keep the limb cold by the application of wet cloths until efficient treatment can be given than to allow partial warming to occur. The actual treatment will consist in using any and every means of preventing too great a rise in the capillary pressure. Whether the use of vaso-constrictors, as suggested above, will be of any avail remains to be seen. They should obviously be given before the part is warmed and once they are acting the parts should not be warmed too slowly. If, on the other hand, vaso-constrictors cannot be given, then the part must be very slowly raised to the normal temperature, for this tends to diminish the degree of vaso-motor paralysis. Elevation of the limb and gentle massage, by aiding the return of blood and diminishing the capillary pressure, will be of value in after-treatment. They will also be of great use as a means of prophylaxis. "

While hoping that vaso-constriction may be tried as a means of relieving the blood pressure and the consequent exudations, the author does not entirely condemn the suggestion that the blocking of the nerve trunks by novocaine might prevent reflex nervous dilation. He is convinced, however, that the dilation in the rabbit is not at all the result of reflex action. In man it may possibly be otherwise.

*Le Gérant* : O. Porée. Paris — Imp. Lahure, 9, rue de Fleurus.

